# The Worcester General Infirmary

**Published:** 1913-11-29

**Authors:** 


### THE WORCESTER GENERAL INFIRMARY.

### Something Attempted at Last.

So long ago as 1910 (The Hospital, April 16, page 89) we called attention to the ridiculously low scale of subscriptions on which the issue of in- and out-patient tickets was based. We showed that every man who gives one guinea to this hospital secures the right of taking at least £5 in value from its funds. The committee took no notice of our recommendation to revise the scale of privileges and to put it on a business footing. Instead of doing so they continued to fritter away invested capital by making good the growing deficiency which necessarily followed the upholding of a system so ruinous as that which prevailed at Worcester. In August last a crisis was reached, and the governors then actually decided by 17 votes to 7 to still further cripple the efficiency of the hospital by reducing the number of the medical, nursing, and domestic staff, and the salaries of the chaplain, matron, secretary, and dispenser.

In The Hospital of August 16 last, page 576, we condemned this policy, and called upon the committee to resign, or, failing this, for the more representative of Worcester citizens to call a special meeting to save this hospital from the ruin with which it was threatened by the inertia of the committee. The matter was taken up in the local press, and as a result a special sub-committee was appointed to consider the best means of increasing the income of the infirmary. The report of this sub-committee was considered at a meeting of the board on the 17th instant, when it was determined to summon a special meeting of governors on December 1 next to alter the rules with a view to enable the following changes to be carried out from January 1, 1914: (1) The qualification of a governor is to be raised from a subscription of one guinea to two guineas, with the right to receive eight out-patient or one in-patient ticket per annum. This means that every governor who uses an in-patient note will impose upon the infirmary an expenditure of £5 16s. in return for his gift of £2 2s. This illustrates the financial unsoundness of the ticket system, for every gift should at least equal the amount of benefit which is extracted from the infirmary by a self-respecting subscriber. Mr. Ward pointed out that the suggested changes would still leave the committee insolvent.

Secondly: works, societies, individuals, and parishes were given the right to reserve beds in the infirmary on payment of £50 per annum. The average cost of a bed per annum at Worcester on the total ordinary expenditure is £79, so that every society, parish, or individual who takes advantage of this proposal will gain £79 in value for every £50 he contributes. (3) Paying patients are to be received on a payment of £1 11s. 6d. at the beginning of each week's residence in the infirmary, by which means it is estimated that the sum of £750 a year may be added to the hospital revenue. Finally, a special canvassing committee is to be formed to endeavour to support and encourage the efforts of the Hospital Saturday and Sunday Collection Funds and the Hospital Penny Monthly Collection Fund, to extend these agencies to parishes and districts where they do not at present collect, and to arrange for a personal canvass.

Incomplete and timid as these recommendations are we welcome them because the committee has at last tried to save the Worcester Infirmary from financial ruin. As will be seen, with the exception of the proposal to admit paying patients, which promises to be self-supporting, the heavy drain resulting from the continuance of the ticket system cannot fail to necessitate a reconsideration of these proposals within a few years, should they be approved at the meeting of governors. We hope the governors will read the report of the proceedings of the committee, as given in the *Worcester Daily Times* of the 18th instant, and note the deliberate attempt at further opposition threatened by some members present by voting and speaking in favour of draining the resources of the Worcester Infirmary to the utmost. We hope some real men and women will attend the meeting on December 1 fully prepared to deal with all obstructors.

